# Supplementary material for: Molecular and Genetic Mechanisms of Spinal Stenosis Formation: Systematic Review
Source: Int J Mol Sci. 2022 Nov 3;23(21):13479. doi: 10.3390/ijms232113479 (PMC9658491; doi:10.3390/ijms232113479)
Supplement: Supplementary file 1 [file ijms-23-13479-s001.zip › ijms-1879865-supplementary.pdf]

Table S1. Complete list of included manuscripts.

Sections of the manuscript	Criterion	Description	Reference
Studies containing information about the spread of diseases that cause spinal stenosis and included in the sections "Epidemiology"	Disease	Ossification of the posterior longitudinal ligament	7
		Ossification of the posterior longitudinal ligament	9
		Ossification of the posterior longitudinal ligament	10
		Ossification of the posterior longitudinal ligament	15
		Ossification of the ligamentum flavum	19
		Facet joint osteoarthritis	34
		Disc herniation	50
		Achondroplasia	57
Studies containing information about genes whose mutations contribute to the development of spinal stenosis and included in the sections “Genetics”	Gene	BMP2	17
		TGF-β1	20
		TGF-β1	21
		BMP2	22
		BMP2	23
		TGF-β1	28
		TGF-β	29
		GNAS	30
		TGF-β1	40
		TGF-β1	42
		TGF-β1	43
		COL1A1	44
		FOXC1	45
		COL1A1 and COL9A2	47
		COL1A1	49
		COL1A2	51
		COL1A1	52
		COL1A1	53
		COL1A2	54
		FGFR3	55
		FGFR3	56
		FGFR3	58
		FGFR3	62
		FGFR3	63
		FGFR3	64
		FGFR3	65
		FGFR3	66
		FGFR3	67
		FGFR3	68
		FGFR3	69
		FGFR3	70
Studies that include information about the biochemical processes involved in the formation of spinal stenosis and included in the sections	Protein	TGF-β	16
		BMP-SMAD	18
		TGF-β	26
		TGF-β	27
		TGF-β	28
		TGF-β1	31
		TGF-β1	32
		TGF-β1	33

"Molecular Mechanism"		Wnt/β-catenin		36
		TGF- β		37
		TGF-β/BMP		38
		TGF-β, SMAD		39
		β-catenin		41
		FGFR3		60
		FGFR3		61
Studies containing information on the characteristics of stenosis caused by biochemical processes in the described diseases, and included in the sections "Stenosis"	Disease/ Characteristics of stenosis	Ossification of the posterior longitudinal ligament	Central, isolated, monosegmental stenosis of the cervical spine	8
				12
				13
			Central, isolated, monosegmental stenosis of the thoracic spine	11
				14
		Ossification of the ligamentum flavum	Central, isolated, monosegmental stenosis of the thoracic and lumbar spine	24
		Achondroplasia	Central, polysegmental tandem stenosis	25
		Facet joint osteoarthritis	Foraminal, isolated, polysegmental stenosis of the lumbar spine	35
		Disc herniation	Central, isolated, monosegmental stenosis of the lumbar spine	50
		Achondroplasia	Central, polysegmental tandem stenosis	59

Table S2. Complete list of full-text articles excluded.

List of excluded full-text articles, with reasons for exclusion		
**№**	**Article metadata**	**Reason for exclusion**
1	Fujimori T, Le H, Hu SS, et al. Ossification of the posterior longitudinal ligament of the cervical spine in 3161 patients: a CT-based study. *Spine (Phila Pa 1976).* 2015;40(7):E394–E403.	Did not meet inclusion criteria
2	Jin H, van't Hof RJ, Albagha OM, Ralston SH. Promoter and intron 1 polymorphisms of COL1A1 interact to regulate transcription and susceptibility to osteoporosis. *Hum Mol Genet.* 2009;18(15):2729–2738.	Did not meet inclusion criteria
3	Kawaguchi Y, Furushima K, Sugimori K, Inoue I, Kimura T. Association between polymorphism of the transforming growth factor-beta1 gene with the radiologic characteristic and ossification of the posterior longitudinal ligament. *Spine (Phila Pa 1976).* 2003;28(13):1424–1426.	Did not meet inclusion criteria
4	Moon BJ, Kuh SU, Kim S, Kim KS, Cho YE, Chin DK. Prevalence, Distribution, and Significance of Incidental Thoracic Ossification of the Ligamentum Flavum in Korean Patients with Back or Leg Pain : MR-Based Cross Sectional Study. *J Korean Neurosurg Soc.* 2015;58(2):112–118.	Required data not provided
5	Alcaraz MJ, Megías J, García-Arnandis I, Clérigues V, Guillén MI. New molecular targets for the treatment of osteoarthritis. *Biochem Pharmacol. 2010*;80(1):13–21.	Did not meet inclusion criteria
6	Bonewald LF, Dallas SL. Role of active and latent transforming growth factor beta in bone formation. *J Cell Biochem.* 1994;55(3):350–357.	Did not meet inclusion criteria
7	Chitty LS, Griffin DR, Meaney C, et al. New aids for the non-invasive prenatal diagnosis of achondroplasia: dysmorphic features, charts of fetal size and molecular confirmation using cell-free fetal DNA in maternal plasma. *Ultrasound Obstet Gynecol.* 2011;37(3):283–289.	Did not meet inclusion criteria

8	Cutroneo KR. TGF-beta-induced fibrosis and SMAD signaling: oligo decoys as natural therapeutics for inhibition of tissue fibrosis and scarring. *Wound Repair Regen.* 2007;15 Suppl 1:S54–S60.	Did not meet inclusion criteria
9	Blaney Davidson EN, van der Kraan PM, van den Berg WB. TGF-beta and osteoarthritis. *Osteoarthritis Cartilage.* 2007;15(6):597–604.	Did not meet inclusion criteria
10	Blaney Davidson EN, Vitters EL, van der Kraan PM, van den Berg WB. Expression of transforming growth factor-beta (TGFbeta) and the TGFbeta signalling molecule SMAD-2P in spontaneous and instability-induced osteoarthritis: role in cartilage degradation, chondrogenesis and osteophyte formation. *Ann Rheum Dis.* 2006;65(11):1414–1421.	Did not meet inclusion criteria
11	Dünker N, Krieglstein K. Targeted mutations of transforming growth factor-beta genes reveal important roles in mouse development and adult homeostasis. *Eur J Biochem*. 2000;267(24):6982–6988.	Did not meet inclusion criteria
12	Epstein N. Ossification of the cervical posterior longitudinal ligament: a review. *Neurosurg Focus.* 2002;13(2):ECP1.	Did not meet inclusion criteria
13	Frank S, Madlener M, Werner S. Transforming growth factors beta1, beta2, and beta3 and their receptors are differentially regulated during normal and impaired wound healing. *J Biol Chem.* 1996;271(17):10188–10193.	Did not meet inclusion criteria
14	Cortese A, Vegezzi E, Lozza A, et al. Diagnostic challenges in hereditary transthyretin amyloidosis with polyneuropathy: avoiding misdiagnosis of a treatable hereditary neuropathy. *J Neurol Neurosurg Psychiatry.* 2017;88(5):457–458.	Letter to the editor
15	Wang H, Yang ZH, Liu DM, Wang L, Meng XL, Tian BP. Association between two polymorphisms of the bone morpho-genetic protein-2 gene with genetic susceptibility to ossification of the posterior longitudinal ligament of the cervical spine and its severity. *Chin Med J (Engl).* 2008;121(18):1806–1810.	Did not meet inclusion criteria
16	Jiang F, Liu GS, Dusting GJ, Chan EC. NADPH oxidase-dependent redox signaling in TGF-β-mediated fibrotic responses. *Redox Biol.* 2014;2:267–272.	Did not meet inclusion criteria
17	Nguyen C, Papelard A, Schnitzler A, Mangione P, Poiraudeau S, Rannou F. Congenital lumbar spinal stenosis associated with Marfan syndrome. *Joint Bone Spine.* 2012;79(2):199–200.	Letter to the editor
18	Miyazawa K, Shinozaki M, Hara T, Furuya T, Miyazono K. Two major Smad pathways in TGF-beta superfamily signalling. *Genes Cells.* 2002;7(12):1191–1204.	Required data not provided
19	Kamiya M, Harada A, Mizuno M, Iwata H, Yamada Y. Association between a polymorphism of the transforming growth factor-beta1 gene and genetic susceptibility to ossification of the posterior longitudinal ligament in Japanese patients. *Spine (Phila Pa 1976).* 2001;26(11):1264–1266.	Did not meet inclusion criteria
20	Karabekir HS, Yildizhan A, Atar EK, Yaycioglu S, Gocmen-Mas N, Yazici C. Effect of ligamenta flava hypertrophy on lumbar disc herniation with contralateral symptoms and signs: a clinical and morphometric study. *Arch Med Sci.* 2010;6(4):617–622.	Did not meet inclusion criteria
21	Pluijm SM, van Essen HW, Bravenboer N, et al. Collagen type I alpha1 Sp1 polymorphism, osteoporosis, and intervertebral disc degeneration in older men and women. *Ann Rheum Dis.* 2004;63(1):71–77.	Required data not provided
22	Garfin SR. A 50-year-old woman with disabling spinal stenosis. *JAMA.* 1995;274(24):1949–1954.	Conference materials
23	Ko S, Vaccaro AR, Lee S, Lee J, Chang H. The prevalence of lumbar spine facet joint osteoarthritis and its association with low back pain in selected Korean populations. *Clin Orthop Surg.* 2014;6(4):385–391.	Required data not provided
24	L'Hôte CG, Knowles MA. Cell responses to FGFR3 signalling: growth, differentiation and apoptosis. *Exp Cell Res.* 2005;304(2):417–431.	Required data not provided
25	Li JM, Zhang Y, Ren Y, et al. Uniaxial cyclic stretch promotes osteogenic differentiation and synthesis of BMP2 in the C3H10T1/2 cells with BMP2 gene variant of rs2273073 (T/G). *PLoS One.* 2014;9(9):e106598.	Required data not provided

26	Matsunaga S, Sakou T. Ossification of the posterior longitudinal ligament of the cervical spine: etiology and natural history. *Spine (Phila Pa 1976).* 2012;37(5):E309–E314.	Required data not provided
27	Kalb S, Martirosyan NL, Kalani MY, Broc GG, Theodore N. Genetics of the degenerated intervertebral disc. *World Neurosurg.* 2012;77(3-4):491–501.	Did not meet inclusion criteria
28	Suri P, Rainville J, Kalichman L, Katz JN. Does this older adult with lower extremity pain have the clinical syndrome of lumbar spinal stenosis? *JAMA*. 2010;304(23):2628–2636.	Conference materials
29	Kawaguchi H, Kurokawa T, Hoshino Y, Kawahara H, Ogata E, Matsumoto T. Immunohistochemical demonstration of bone morphogenetic protein-2 and transforming growth factor-beta in the ossification of the posterior longitudinal ligament of the cervical spine. *Spine (Phila Pa 1976).* 1992;17(3 Suppl):S33–S36.	Did not meet inclusion criteria
